# Peri-operative complications in pediatric and adolescent shoulder arthroscopy

**DOI:** 10.1007/s11832-014-0595-y

**Published:** 2014-06-01

**Authors:** Eric W. Edmonds, Laura W. Lewallen, Michael Murphy, Diane Dahm, Amy L. McIntosh

**Affiliations:** 1 Division of Orthopaedic Surgery, Rady Children’s Hospital and Health Center, 3030 Children’s Way, Suite 410, San Diego, CA 92123 USA; 2 Department of Orthopedic Surgery, University of California San Diego, San Diego, CA USA; 3 Department of Orthopaedic Surgery, Mayo Clinic, Rochester, MN USA

**Keywords:** Shoulder arthroscopy, Acute complications, Pediatric, Children, Adolescents

## Abstract

**Background:**

Shoulder arthroscopy is not common in the pediatric and adolescent population, but the frequency may be on the rise. The purpose of the study was to determine the incidence of acute complications of arthroscopic shoulder surgery in children and adolescents.

**Methods:**

A retrospective, cross-sectional review was performed identifying patients aged 18 years or less who underwent an arthroscopic shoulder procedure from 1997 to 2009 at Institution 1 and 2007 to 2010 at Institution 2. Exclusion criteria included open procedures and missing records. Demographic and surgical data were collected, including intra-operative and post-operative complications during the first 6 months. The complications were divided into minor (no secondary treatment) and major (secondary treatment rendered).

**Results:**

Two hundred children, mean age 15.9 years, met criteria and 73 % were boys. All procedures were performed under general anesthesia, but 51 % included inter-scalene regional anesthesia. There were 16 (8.0 %) total complications recorded. Major complications occurred in five (2.5 %) patients, including two tendinitis/bursitis requiring injections, one broken pain pump catheter requiring an accessory incision to retrieve, one pain control readmission, and one laceration of the cephalic vein requiring ligation. Minor complications occurred in 11 (5.5 %) patients, including allergic reactions, transient dysesthesias, headaches, bronchitis, syncope, transient hypotension, and uvula swelling.

**Conclusion:**

Although we found no seriously deleterious outcomes, it is important to recognize that an additional service was rendered for 2.5 % of children undergoing shoulder arthroscopy. The events that did occur may be preventable and this study should serve as a baseline to improve quality and safety of shoulder arthroscopy in the pediatric population.

## Introduction

There are an estimated 7 million high school students participating in athletics annually with an overall rate of severe injury of 0.93 injuries per 1,000 athletic exposures [1]. Although the knee was the most commonly injured body part, this same epidemiology study found that 10.9 % of all the injuries occurred at the shoulder with a significant portion requiring surgical intervention.

There are also reports of surgical intervention in the pre-teenage population, particularly for instability, labral pathology and shoulder impingement [2–4]. Shoulder arthroscopy has also been described in even the very young child. Brachial plexus birth palsy can be treated with secondary joint corrections, and muscle transpositions that are often assisted by arthroscopic visualization [5].

In 1986, a large-scale survey was performed requesting surgeons to report their arthroscopy cases and associated complications [6]. The final report included knee, shoulder, ankle, elbow, and wrist arthroscopy and found a 0.56 % incidence of complications within a cohort of nearly 400,000 reported arthroscopy cases. This report included predominately adult patients, but the highest complication rate was noted in anterior capsulorrhaphy of the shoulder (5.3 %).

Most reports on shoulder arthroscopy in referencing complications merely note an increased incidence of failure of surgery in the under 20-year old cohort [2, 7, 8]. To the best of our knowledge, there is no previous report on children evaluating complications in the acute time period following shoulder arthroscopy. In the modern era of defining quality, safety, and value to care for patients and their pathology it is important to understand a baseline using modern arthroscopic technology. With a null hypothesis that children would not have different outcomes compared to adults, the purpose of this study to determine the incidence of acute complications of arthroscopic shoulder surgery in children.

## Materials and methods

After obtaining institutional review board approval, a retrospective review was performed at two different tertiary care medical centers in two distinct geographic locations of the United States. Both institutions evaluated patients under the age of 18 years by identifying CPT codes associated with an arthroscopic shoulder procedure. This age criteria was utilized because, although proximal humerus physeal closure may begin at age 13 in girls and age 14 in boys, they may remain open until 18 and 20 years, respectively [9, 10]. Institution 1 is a free-standing children’s hospital and included patients from 2007 to 2010. Institution 2 is not limited by age and included children from 1997 to 2009. Patients were treated by multiple surgeons. Exclusion criteria at both institutions included open procedures and missing records.

Demographic and surgical data were collected by chart review of both institutions. Demographics included gender, age, and body-mass index (BMI). Pre-operative medication utilization—especially, birth-control (OCP), non-steroidal anti-inflammatory drugs (NSAIDS), and disease-modifying antirheumatic drugs (DMARDS)—were recorded. Pre-surgical medical co-morbidities such as diabetes, asthma, blood clotting disorders, and rheumatologic disorders were recorded.

Anesthesia type was also recorded for each patient: general, regional, spinal, local, and combinations. The patient positioning at the time of surgery was recorded: beach chair versus lateral decubitus. The in-flow pump pressure during arthroscopy was recorded by specific mmHg versus gravity. Operative times were determined from the anesthesia records and utilization and type of deep venous thrombosis (DVT) prophylaxis and peri-operative antibiotic utilization were recorded. The surgery performed, determined by CPT code, as well as the post-operative management, were recorded.

Complications were also recorded during the intra-operative course and the post-operative first 6 months. The initial assessment of the record tried to identify the following complications, but included the category of “other”: instrument breakage, nerve injury (including spinal headaches), vascular injury, anesthetic complication, hemarthrosis, thromboembolic events, infections, other wound complications, complex regional pain syndrome, arthrofibrosis, and death.

The complications were divided into groups: minor (no secondary treatment rendered) and major (a secondary treatment rendered either at index surgery or post-operatively). SPSS v. 12 was utilized for all statistical analyses. For each variable, the mean and 95 % confidence interval were calculated for each group.

## Results

Two hundred children (73 % boys), mean age of 15.9 years (range 1–18 years old) and mean BMI of 24.4, met criteria. During the study period, there were 237 surgeries performed; five did not meet follow-up criteria (2.1 % loss of follow-up for the study), 31 were not arthroscopic procedures, and one had a missing chart. Regarding age, there were only three infants in the cohort, the remainder were older children or adolescents. Seventy-five (51 %) of the boys were 16 years and younger, and 13 (24 %) of the girls were 14 years and younger, representing a total of 44 % of the entire cohort that were most likely skeletally immature (by age criteria) at the time of surgery.

In comparison between the two institutions, Institution 1 had 95 children that met criteria with a mean age of 15.7 years and a mean BMI of 25.2. Institution 2 had 105 children that met criteria with a mean age of 16.1 years and a mean BMI of 23.7.

Pre-operatively, 2 % of the patients were using birth-control and 12.5 % were using NSAIDs. There were three children with brachial plexus palsy in the cohort and 27 (13.5 %) with asthma. DVT prophylaxis was not used in any of the patients, but in contrast, peri-operative antibiotics were used in all cases (except two unknown with unavailable data).

Although all procedures were performed under general anesthesia, 51 % had inter-scalene regional anesthesia, 13 % utilized an indwelling glenohumeral catheter to allow continuous delivery of local anesthetic medication, and 41 % had local anesthesia injected peri-incisionally and intra-articularly. One hundred and forty-four (72 %) had the surgery in a lateral decubitus position. The mean surgical time (from the patient entering the operating room to exiting the room) for both institutions was 104 min, and the majority of surgery involved anterior or superior labral pathology and instability (87.5 %), compared to posterior labral pathology and instability (8.5 %), or other procedures including partial articular-sided tendon avulsion debridement, subacromial decompression, loose bodies, and distal clavicle resection (4 %). Only 26 % of the patients had their surgery utilizing gravity flow, and the others had a pump maintaining a pressure of 30 mmHg (at Institution 1) and a flow of 100 mL/min (at Institution 2).

There were 16 (8.0 %) total complications recorded. Major complications occurred in five (2.5 %) patients and minor complications in 11 (5.5 %) patients. Major complications included two children with tendinitis/bursitis that required subacromial steroid injections in order to progress with therapy, one broken pain pump catheter that required an accessory portal to retrieve at the time of surgery from its intra-articular position, one readmission for pain control, and one laceration of the cephalic vein that required ligation. The minor complications included three allergic reactions [contact dermatitis (metal and iodine) and an antibiotic reaction], two transient hand dysesthesias, two post-operative headaches (defined as "muscular" by the primary physician), and one of each (identified within 2 weeks following surgery) bronchitis, syncope, transient hypotension, uvula swelling. There were no deaths, septic arthritis, DVT, or CRPS (complex regional pain syndrome) reported during the first 6-month post-operative period. Also, no chondrolysis was seen in the 13 % of patients that had indwelling glenohumeral catheters to allow continuous delivery of local anesthetic medication post-operatively. None of the complications required a return to the operating room.

## Discussion

Our study of children undergoing shoulder arthroscopy compares well with another multicenter instability registry that demonstrated 83 % anterior instability and 10 % posterior instability seen at the time of surgery, which is similar to our findings of 87.5 and 8.5 %, respectively [11]. These combined data suggest that our cohort may well represent the patients seen for most surgeons treating instability.

A large study evaluating arthroscopy complications on a primarily adult population found shoulder complications as high as 5.3 % [6]. In our cohort, a direct comparison of complications is difficult, because the types of complications seen were quite different [6, 12–14]. Whereas, the adult population is at risk for nerve injury, infection, and DVT, our young cohort appears to be primarily at risk for issues not recorded in these previous adult studies. Even our major complications were relatively minor, given that they required a subsequent injection in-clinic or an additional arthroscopy portal at the time of index procedure. However, it is important to recognize that an additional service was rendered for 2.5 % of children undergoing shoulder arthroscopy.

It is important to note that peri-operative antibiotics were provided for at least 99 % of our cohort, and we had no recorded septic arthritis or superficial wound infections. Conjecture regarding why antibiotics were provided could range from thoughtful utilization because implants were utilized to mere system-based prophylaxis related to “Pay for Performance” measures. Unfortunately, with multiple surgeons involved, it is unclear as to the actual reason antibiotics were administered. It is interesting to note that perhaps blind utilization of antibiotics is not without risk, since a couple of the minor complications were directly related to the antibiotic or cleaning of the skin during surgical preparation. Furthermore, no DVT prophylaxis was utilized (mechanical or pharmacologic) and no evidence of thromboembolic event was recorded.

Beach-chair and lateral decubitus positions were utilized equally within our cohort with no association with complication noted between groups. Similarly, there appeared to be no association with complication between children using OCPs or NSAIDs, nor was there an association with pre-operative medical comorbidities. Other than allergic reactions, no complication carried >1 % risk, and most occurred with only 0.5 % risk of occurrence.

Apropos clinical pathway measures that may be implemented to augment the care of this young surgical population include standardization of surgical and anesthetic approach. Standardizing to position of surgery and implants should reduce risks of many minor complications seen in this cohort that may be related to positioning and padding. A review paper recently published highlights this need to consider the size constraints of the pediatric shoulder and to further remember that children are not just small adults [15]. It also reduces the risk that surgical team members may be unfamiliar with surgical setup. Moreover, many of the minor complications were related to anesthesia, and it seems that further study is required to determine the best pathways to minimize risk, in this regard.

Many of the complications seemed to unfold during the first 1–2 weeks, so a possible clinical pathway measure could be to have the anesthesiologist involved with the case, call the family a day or two after surgery. This phone call can elicit any issues with regional anesthesia and help guide pain management which may prevent issues with headache or readmission for pain control. This practice was initiated by the anesthesiologists at Institution 1 with great success to support the family with symptoms related to anesthesia, as well as to guide pain management (since these anesthesiologists comprise the majority of the physician pool for the pain service). Since implementation, there have been no re-admissions for pain control and no post-operative headaches recorded.

A limitation to this study is the retrospective design, which allows for the possibility that complications may have occurred and were not recorded. Furthermore, since there is a low rate of intra-articular shoulder pathology in the skeletally immature population, there is a corresponding low rate of surgery to treat children [16]. Therefore, the size of our cohort may be considered small by adult incidence standards [6], but it is still a fairly large, multicenter cohort considering this innate limitation to studying this particular population.

The results of this study should provide data to institute clinical pathways to reduce the risk of complication with shoulder arthroscopy in children. Although there is low risk overall for these procedures with an incidence of 2.5 % complications requiring a secondary intervention, there is clearly room to improve results regarding early complications.
